# Prostaglandin E receptor 4 (EP4) promotes colonic tumorigenesis

**DOI:** 10.18632/oncotarget.5589

**Published:** 2015-09-10

**Authors:** Jian Chang, Jean Vacher, Bing Yao, Xiaofeng Fan, Bixiang Zhang, Raymond C. Harris, Ming-Zhi Zhang

**Affiliations:** ^1^ Department of Medicine, Vanderbilt University, Nashville, Tennessee, USA; ^2^ Cancer Biology, Vanderbilt University, Nashville, Tennessee, USA; ^3^ Départment of Médecine, Clinical Research Institute of Montreal, Université de Montréal, Montreal, Quebec, Canada; ^4^ Hepatic Surgery Center, Tongji Hospital, Tongji Medical College, Huazhong University of Science & Technology, Wuhan, China; ^5^ Hepatobiliary Surgery Department, Wuhan No.1 Hospital, Wuhan, China; ^6^ Department of Molecular Physiology and Biophysics, Vanderbilt University, Nashville, Tennessee, USA; ^7^ Jiangsu Center for The Collaboration and Innovation of Cancer Biotherapy, Cancer Institute, Xuzhou Medical College, Xuzhou, China

**Keywords:** cyclooxygenase-2, myeloid cells, polarization, tumor growth

## Abstract

Colorectal cancer (CRC) continues to be a major cause of morbidity and mortality. Although the factors underlying CRC development and progression are multifactorial, there is an important role for tumor-host interactions, especially interactions with myeloid cells. There is also increasing evidence that cyclooxygenase-derived prostaglandins are important mediators of CRC development and growth. Although prevention trials with either nonselective NSAIDs or COX-2 selective agents have shown promise, the gastrointestinal or cardiovascular side effects of these agents have limited their implementation. The predominant prostaglandin involved in CRC pathogenesis is PGE_2_. Since myeloid cells express high levels of the PGE_2_ receptor subtype, EP_4_, we selectively ablated EP_4_ in myeloid cells and studied adenoma formation in a mouse model of intestinal adenomatous polyposis, *Apc*^Min/+^ zmice. *Apc*^Min/+^mice with selective myeloid cell deletion of EP_4_ had marked inhibition of both adenoma number and size, with associated decreases in mTOR and ERK activation. Either genetic or pharmacologic inhibition of EP_4_ receptors led to an anti-tumorigenic M1 phenotype of macrophages/dendritic cells. Therefore, PGE_2_-mediated EP_4_ signaling in myeloid cells promotes tumorigenesis, suggesting EP_4_ as a potentially attractive target for CRC chemoprevention or treatment.

## INTRODUCTION

Colorectal cancer (CRC) is one of the most preventable cancers; however it is still the leading cause of cancer death. Primary prevention remains the best approach to reducing overall morbidity and mortality. Arachidonic acid metabolism by the cyclooxygenase (COX) pathway has been implicatesd as an important contributor to CRC development and growth. COX is the rate-limiting enzyme in the metabolism of arachidonic acid to prostaglandin G_2_/H_2_ (PGG_2_/H_2_), which serves as the precursor for subsequent metabolism by prostaglandin and thromboxane synthases. Two isoforms of COX exist in mammals, “constitutive” COX-1 and inflammatory-mediated and glucocorticoid-sensitive COX-2. There is a clear molecular link between COX-2 and COX-2-derived PGE_2_ and CRC progression [1, 2].

In the last two decades, inhibition of COX-2-derived PGE_2_ by traditional NSAIDs or selective COX-2 inhibitors has been proven to be successful in reducing the number and burden of colorectal polyps in humans and animal models of familial adenomatous polyposis (FAP) patients. However, increased gastrointestinal side effects due to long-term use of traditional NSAIDs and increased cardiovascular events due to chronic use of selective COX-2 inhibitors limit their utility in chemoprevention/chemotherapy of CRC [3-8]. Therefore, new strategies to inhibit the COX-2 pathway with fewer associated side effects are needed for chemoprevention/chemotherapy of CRC.

Tumor-host interactions play a key role in the development and progression of cancers. The solid tumor stromal microenvironment consists of infiltrating immune cells (macrophages and lymphocytes), fibroblasts, myofibroblasts, adipocytes, endothelial cells and pericytes, as well as a variety of extracellular matrix components [9-11]. The role of tumor associated macrophages is of particular interest in CRC development and growth, because macrophages can exhibit distinctly different functional phenotypes, broadly characterized as a pro-inflammatory (M1 or “classically activated”) phenotype, which is anti-tumorigenic and a tissue reparative (M2 or “alternatively activated”) phenotype, which is pro-tumorigenic [12]. Myeloid cells have been proposed to promote tumor initiation, tumor growth, metastasis and immunomodulation.

In both human sporadic colorectal adenomas and intestinal adenomas in *Apc*^Min/+^ mice, COX-2 is highly expressed in macrophages [13, 14]. PGE_2_ signals through four distinct G protein coupled receptors- EP_1_ through EP_4_. EP_1_ is coupled primarily to G_q_/G_11_ and EP_3_ to G_i_, while EP_2_ and EP_4_ are primarily coupled to G_s_. EP_4_ is the predominant prostaglandin receptor in macrophages [15], and EP_4_ activation in macrophages inhibits macrophage cytokine and chemokine release [16-19]. In addition, COX-2 and EP_4_ are also expressed in other immune cells. In the present study, we determined that selective deletion of EP_4_ receptors in myeloid cells effectively inhibited intestinal adenoma development and growth. Furthermore, either genetic or pharmacologic inhibition of EP_4_ receptors led to inhibition of ERK and mTOR pathways in adenomas in association with decreased M2 and increased M1 phenotypic macrophages. These findings suggest an important role for myeloid cell EP_4_ receptors in regulation of colorectal tumorigenesis and identify EP_4_ receptor as a possible target for prevention and/or therapy for colorectal cancer.

## RESULTS

### Deletion of myeloid EP_4_ receptors led to marked inhibition of adenoma development and growth in Apc^*Min/+*^ mice

**Figure 1:**
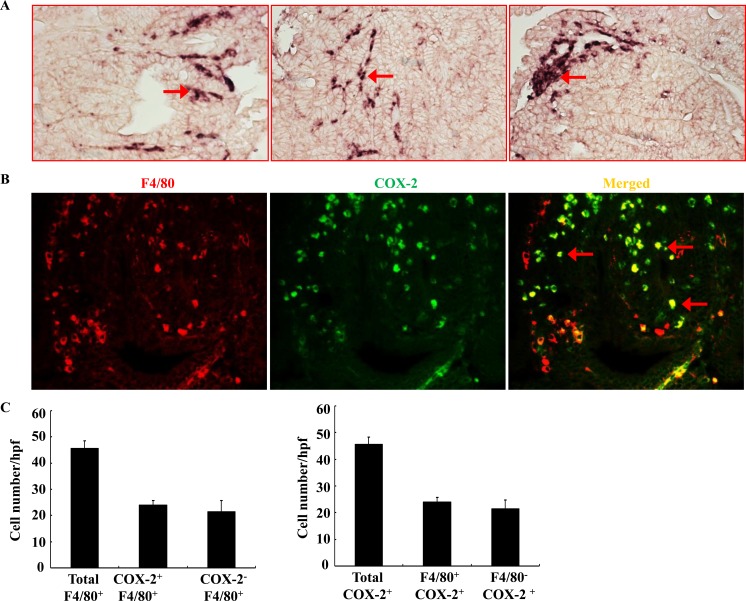
Localization of COX-2 in adenoma from *Apc*^**Min/+**^ mice **A.** *In situ* hybridization showed that COX-2 mRNA was highly expressed in adenoma stroma (arrows). Each panel was from a different adenoma. Original magnification: x160. **B.** Double fluorescent staining showed localization of F4/80 (red, a marker of macrophage/dendritic cells) and COX-2 (green). Arrows indicate COX-2 expressing macrophages/dendritic cells. Original magnification: x 400. **C.** Quantitative data indicated that approximately half of F4/80-positive macrophages/dendritic cells expressed COX-2 and approximately half of COX-2-positive cells were also F4/80-positive.

As noted, COX-2 has been previously reported to be highly expressed in stromal cells in *Apc*^Min/+^ mouse intestinal adenomas and in human sporadic colorectal adenomas [13, 14]. However, COX-2 deletion in myeloid cells did not affect intestinal tumorigenesis in *Apc*^Min/+^ mice [20]. Using *in situ* hybridization, we confirmed that COX-2 mRNA was highly expressed in adenoma stromal cells of *Apc*^Min/+^ mouse (Figure 1A). Using double immunofluorescent staining, we further found that about ∼ 50% of adenoma macrophages/dendritic cells (F4/80 positive cells) expressed COX-2. Similarly, about ∼ 50% of COX-2-positive stromal cells were F4/80-positive (Figure 1B & 1C). Therefore, only half of COX-2-positive cells in adenoma stroma were macrophages/dendritic cells in *Apc*^Min/+^ mice. The non-macrophage/dendritic cell COX-2 positive stromal cells may include endothelial cells, lymphocytes and other cell types.

**Figure 2:**
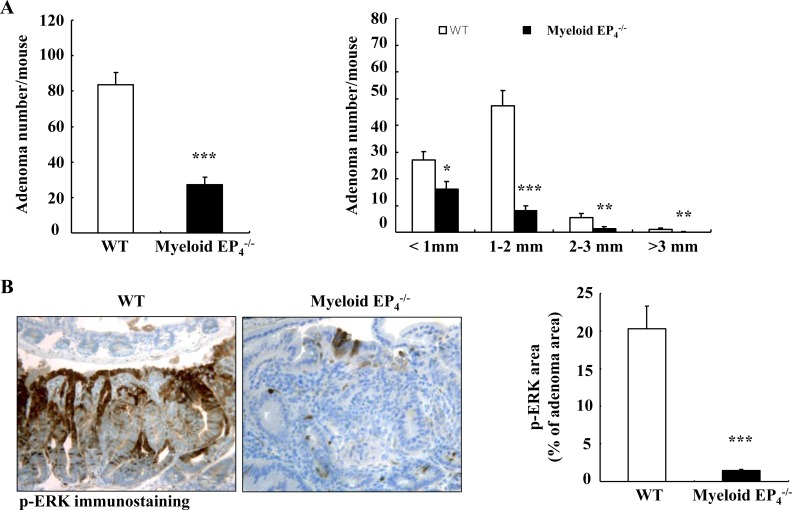

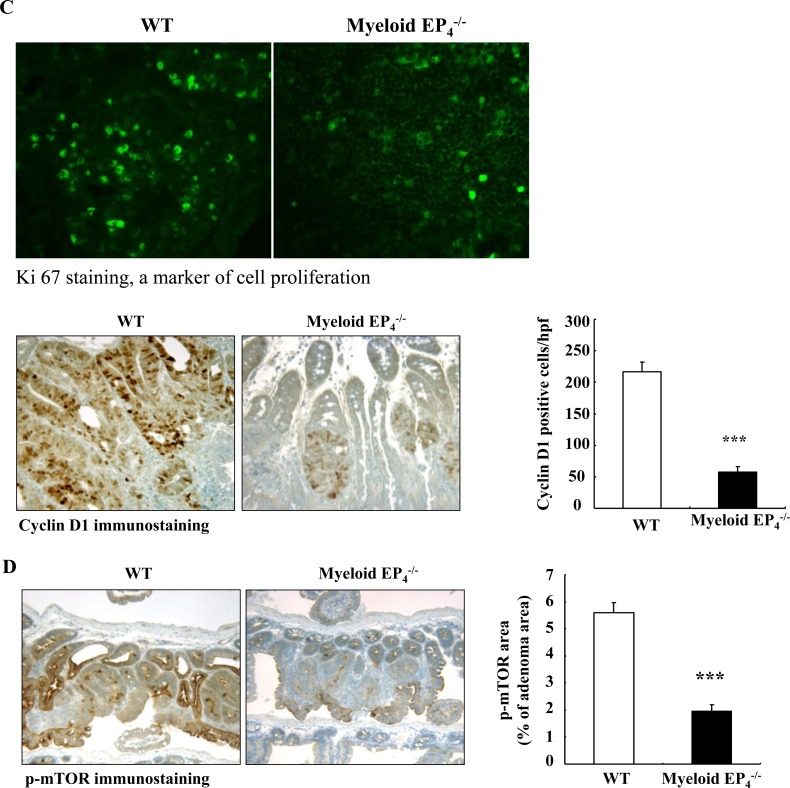

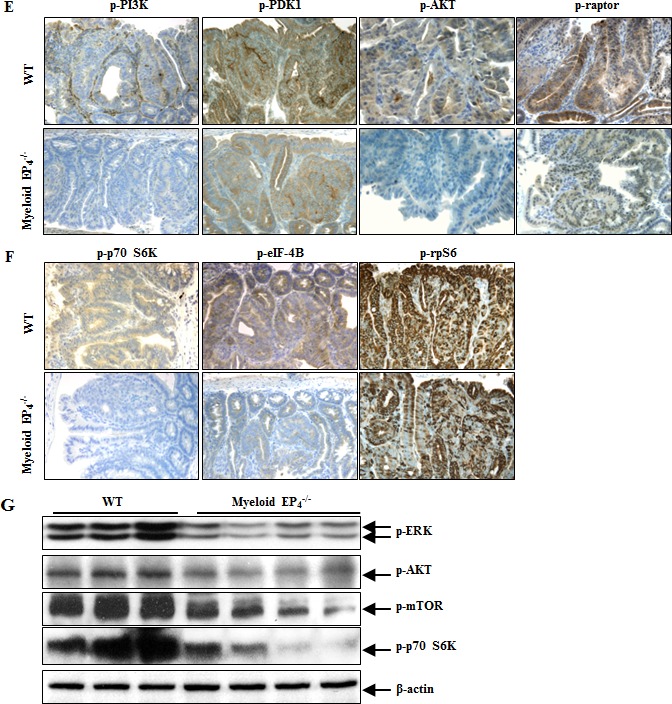
Myeloid cell prostaglandin EP_4_ receptors promoted tumorigenesis in *Apc*^**Min/+**^ mice in association with activation of ERK and mTOR signaling pathways **A.** Deletion of myeloid cell EP_4_ receptors significantly reduced *Apc*^Min/+^ mouse intestinal adenoma multiplicity (***P < 0.001, *n* = 15 in wild type group and *n* = 14 in myeloid cell EP_4_^−/−^ group) and sizes (*P < 0.05, **P < 0.01, ***P < 0.001). **B.** Immunostaining indicated that expression of phosphorylated ERK, apparent in most tumor epithelial cells in wild type *Apc*^Min/+^ mice, was dramatically reduced in myeloid cell EP_4_^−/−^ *Apc*^Min/+^ mice (***P < 0.001, *n* = 4 in each group). **C.** Cyclin D1 was primarily localized to adenoma epithelial cell nuclei, and its expression markedly decreased in myeloid cell EP_4_^−/−^ *Apc*^Min/+^ mice (***P < 0.001, *n* = 4 in each group). The staining density of Ki67, a marker of cell proliferation, was decreased in myeloid cell EP_4_^−/−^ *Apc*^Min/+^ mice. **D.** Immunostaining indicated that phosphorylated mTOR was highly expressed in tumor epithelial cells and that its expression was dramatically inhibited in myeloid cell EP_4_^−/−^ *Apc*^Min/+^ mice (***P < 0.001, *n* = 4 in each group). **E.** and **F.** Deletion of myeloid cell EP_4_ receptors also led to decreases in adenoma phosphorylation levels of PI3K, PDK1, AKT and raptor (E) as well as decreased adenoma phosphorylation levels of p70 S6K, eIF-4B and rpS6. **G.** Immunoblotting determined decreased expression levels of adenoma p-ERK, p-AKT, p-mTOR and p-p70 S6K in myeloid cell EP_4_^−/−^ *Apc*^Min/+^ mice. Original magnification: x160 in all except x 400 for Ki67.

PGE_2_ acts in myeloid cells primarily through activation of prostaglandin EP_4_ receptors [15]. We hypothesized that selective EP_4_ deletion in myeloid cells might have profound effects on tumorigenesis in *Apc*^Min/+^mice because myeloid cell EP_4_ receptors may mediate the actions of PGE_2_ generated by both COX-1 and COX-2 from myeloid cells as well as other cell types (non-myeloid stromal cells and tumor epithelial cells). We generated EP_4_^flox/flox^; *Apc*^Min/+^mice (WT) and CD11b-Cre; EP_4_^flox/flox^ (myeloid cell EP_4_^−/−^) *Apc*^Min/+^mice and sacrificed them at 20 weeks of age. Isolated intestinal myeloid cells from myeloid EP_4_^−/−^ *Apc*^Min/+^mice had decreased EP_4_ mRNA levels (Supplemental Figure S1A). Only male mice had EP_4_ deletion in myeloid cells due to CD11b-Cre insertion into the Y chromosome [21]. We confirmed that the body weights of myeloid cell EP_4_^−/−^ *Apc*^Min/+^ mice were significantly greater than male as well as female WT *Apc*^Min/+^ mice (Supplemental Figure S1B). Deletion of myeloid cell EP_4_ receptors markedly reduced adenoma number and size (adenoma/mouse: 27.3 ± 4.1 *vs*. 83.6 ± 7.0 of WT *Apc*^Min/+^ mice, *P* < 0.0001, *n* = 15 in WT *Apc*^Min/+^ mice and *n* = 14 in myeloid EP_4_^−/−^ *Apc*^Min/+^ mice) (Figure 2A). Colonic adenoma number was comparable between WT and myeloid cell EP_4_^−/−^ *Apc*^Min/+^ mice (colonic adenoma/mouse: 1.44 ± 0.34 *vs*. 1.67 ± 0.37 of WT, *n* = 16). No gender difference was found for adenoma number and size between male and female WT *Apc*^Min/+^ mice (Supplemental Figure S2A).

### Deletion of myeloid cell EP_4_ receptors led to inhibition of the adenoma ERK and PI3K-AKT-mTOR signaling pathways in Apc^*Min/+*^ mice

We utilized immunohistochemistry with quantitative analysis to investigate the potential mechanisms by which myeloid EP_4_ receptors regulated tumorigenesis. Extracellular signal-regulated kinase (ERK) activation plays a key role in PGE_2_-mediated colorectal tumorigenesis [1, 22]. Deletion of myeloid EP_4_ receptors led to markedly decreased adenoma ERK phosphorylation (Figure 2B), in association with inhibition of tumor cell proliferation, as indicated by significantly reduced expression levels of adenoma cyclin D1 and ki67 (Figure 2C) as well as c-Myc (Supplemental Figure S2B).

In colon cancer cells, PGE_2_ also stimulates cell proliferation through activation of the PI3K-AKT-mTOR cascade [23]. Adenomas of myeloid cell EP_4_^−/−^ *Apc*^Min/+^ mice had decreased mTOR phosphorylation (Figure 2D), in association with decreased expression levels of phosphorylated PI3K, PDK1, AKT and raptor (Figure 2E). In addition, adenomas of myeloid cell EP_4_^−/−^ *Apc*^Min/+^ mice also had decreased phosphorylation of downstream targets of mTOR signaling, p70 S6K and eIF-4B (Figure 2F). Furthermore, phosphorylated S6 ribosomal protein (rpS6, Ser235/236), a downstream target of p70 S6K, was also decreased in adenomas of myeloid EP_4_^−/−^ *Apc^Min/+^* mice (Figure 2F). Immunoblotting confirmed the decreased levels of adenoma p-ERK, p-AKT, p-mTOR and p-p70 S6K in myeloid EP_4_^−/−^ *Apc^Min/+^* mice (Figure 2G).

### Deletion of myeloid EP_4_ receptors led to loss of the pro-tumorigenic M2 phenotype for adenoma macrophages/dendritic cells in Apc^*Min/+*^ mice

**Figure 3:**
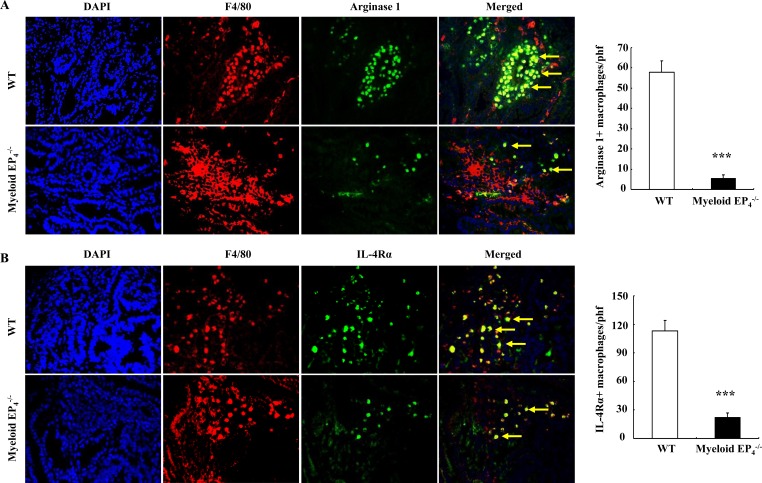
Myeloid cell EP_4_ receptors are essential in polarization and maintenance of an M2 phenotype for macrophages/dendritic cells **A.** Most TAMs expressed arginase 1 (yellow arrows) in wild type *Apc*^Min/+^ mice, but only in a small portion of macrophages/dendritic cells in myeloid cell EP_4_^−/−^ *Apc*^Min/+^ mice (***P < 0.001, *n* = 4 in each group). **B.** IL-4Rα, another marker of M2 macrophages/dendritic cells, was expressed in most macrophages/dendritic cells in wild type *Apc*^Min/+^ mice, but only in a small portion of macrophages/dendritic cells in myeloid cell EP_4_^−/−^ *Apc*^Min/+^ mice (yellow arrows) (***P < 0.001, *n* = 4 in each group). Original magnification: 250 in all.

Arginase 1 is required for macrophage/dendritic cell polarization into an M2 phenotype and is also used as a marker for the M2 phenotype [12, 24]. Arginase 1 was highly expressed in tumor stroma, but undetectable in normal intestine adjacent to adenoma *Apc*^Min/+^ mice (Supplemental Figure S3A). Double fluorescent staining confirmed that these arginase 1 expressing cells were F4/80-positive macrophages/dendritic cells (Supplemental Figure S3B). Both the arginase 1 immunostaining density and the number of arginase 1-positive macrophages/dendritic cells were markedly reduced in myeloid EP_4_^−/−^ *Apc*^Min/+^ mice (arginase 1 positive cells/phf: 5.7 ± 1.6 *vs*. 57.96 ± 5.5 of WT *Apc*^Min/+^ mice, *P* < 0.001, *n* = 6 in each group) (Figure 3A). The number of macrophages/dendritic cells expressing IL-4Rα, another marker for M2 macrophages/dendritic cells, was also markedly reduced in myeloid cell EP_4_^−/−^ *Apc*^Min/+^ mice (IL-4Rα-positive cells/phf: 22.2 ± 4.8 *vs*. 113.2 ± 10.7 of WT *Apc*^Min/+^ mice, *P* < 0.001, *n* = 6 in each group) (Figure 3B).

### Antagonism of EP_4_ receptors polarized macrophages/dendritic cells to an anti-tumorigenic M1 phenotype in Apc^*Min/+*^ mice

**Figure 4:**
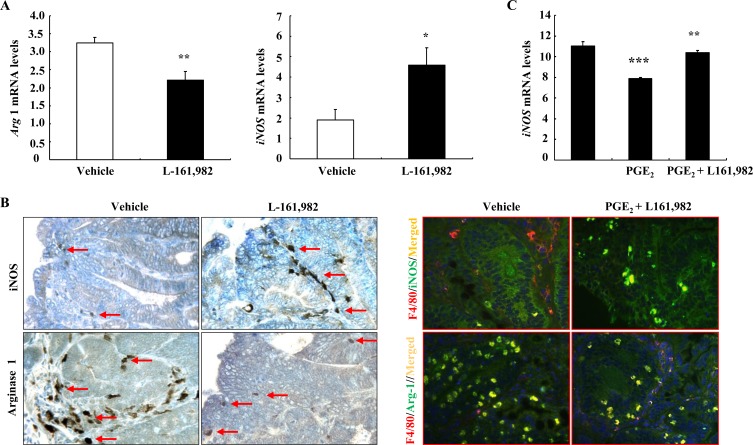
Pharmacologic inhibition of EP_4_ receptors led to alteration of macrophages/dendritic cells from an M2 to an M1 phenotype **A.** Treatment with L-161,982, a selective EP_4_ receptor antagonist for a week led to increased mRNA levels of the M1 marker iNOS, but decreased mRNA levels of the M2 marker arginase 1 (* *P* < 0.05 and ** *P* < 0.01 *vs*. vehicle, *n* = 5). **B.** Immunostaining showed increased iNOS positive stromal cells (M1 marker) but decreased arginase 1 positive stromal cells (M2 marker). Arrows indicate macrophages/dendritic cells expressing either iNOS or Arginase 1. Co-immunostaining showed that EP_4_ antagonism led to increase in F4/80 and iNOS double positive cells (M1 macrophages/dendritic cells) but decrease in F4/80 and arginase-1 (Arg-1) double positive cells (M2 macrophages/dendritic cells). Original magnification: x250 for immunohistochemistry, x400 for co-immunostaining. **C.** In murine macrophage RAW264.7 cells, PGE_2_ treatment led to decreases in iNOS mRNA levels, which were prevented by EP_4_ antagonism (** *P* < 0.01 *vs*. vehicle; *** *P* < 0.001 *vs*. PGE_2_ alone. *N* = 3).

To investigate whether pharmacologic inhibition of the EP_4_ receptor would alter the macrophage/dendritic cell phenotype, WT *Apc*^Min/+^ mice at 16 weeks of age were treated for one week with L-161,982, a selective EP_4_ receptor antagonist, and adenomas were then harvested for analysis. The EP_4_ antagonist decreased adenoma phosphorylation of both ERK and mTOR, inhibited expression of arginase 1 mRNA and protein (Supplementary Figure S4 and Figure 4). In contrast, both mRNA and protein levels of iNOS, a marker of the M1 phenotype, were increased with EP_4_ antagonism (Figure 4A & 4B). Co-immunostaining indicated that EP_4_ antagonism led to decreased number of F4/80 and arginase double positive cells but increased number of F4/80 and iNOS double positive cells (Figure 4B). In addition, L-161,982 treatment led to marked increases in adenoma caspase-3 mRNA and protein levels, indicating an increase in apoptosis (Supplemental Figure S4 B and C). Furthermore, in the murine macrophage cell line, RAW264.7, treatment with PGE_2_ led to decreases in iNOS mRNA levels, which were reversed by pretreatment with L-161,982 (Figure 4C).

## DISCUSSION

Both selective COX-2 inhibitors and global COX-2 deletion have been shown to effectively suppress adenoma development and growth [5, 25, 26], and COX-2 is highly expressed in myeloid cells in both human sporadic colorectal adenomas and intestinal adenomas in *Apc*^Min/+^ mice [13, 14]. Using *in situ* hybridization, we confirmed that COX-2 mRNA was highly expressed in adenoma stroma cells (Figure 1). However, double immunofluorescent staining indicated that COX-2-expressing macrophages/dendritic cells make up only about half of the COX-2-expressing cells in the adenoma stroma. This may explain why COX-2 deletion in the myeloid cell lineage did not affect intestinal tumorigenesis in *Apc*^Min/+^ mice [20].

EP4 receptor expression is increased in colorectal cancer, and expression is correlated to tumor cell growth [27]. PGE2 has been reported to stimulate human colon cancer cell proliferation through an EP4 receptor-mediated PI3K and ERK signaling pathways [27-29]. In murine colon adenocarcinoma CT-26 cells, the anti-proliferative effects of COX inhibition were rescued specifically by an EP4 receptor agonist via PI3K/ERK activation, thus providing a functional link between PGE2-induced cell proliferation and EP4 receptor mediated ERK signaling [30]. Global deletion of the EP4 receptor inhibited colorectal tumorigenesis *in vivo* [31]. Furthermore, antagonism of host EP4 receptors reduces colon cancer metastasis, consistent with involvement of macrophages, a major component of tumor stroma or microenvironment [32]. However, the role of myeloid cell EP4 receptors in colorectal tumorigenesis has not been previously investigated.

The present results indicate that myeloid cell EP4 receptors play an essential role in intestinal adenoma development and growth in *Apc*^Min/+^ mice. We generated *Apc*^Min/+^ mice with selective deletion of EP4 receptors in myeloid cells and found that selective EP4 deletion in myeloid cells effectively reduced adenoma number and size in *Apc*^Min/+^ mice in association with inhibition of adenoma activities of ERK, PI3K, PDK1, AKT, mTOR and its downstream targets, p60 S6K and eIF-4B and rpS6. Theoretically, EP_4_ receptors on myeloid cells can be activated by PGE_2_ generated by COX-2 in myeloid cells themselves in an autocrine pattern or PGE_2_ generated in other stromal cells and in tumor epithelial cells in a paracrine pattern. Furthermore, PGE_2_ generated by COX-1 in all these cells can also activate EP4 receptors on myeloid cells as well. Therefore, myeloid cell EP4 deletion is more effective than myeloid cell COX-2 deletion at inhibiting tumorigenesis.

Deletion of myeloid cell EP4 receptors also led to phenotypic alteration of macrophages/dendritic cells from a pro-tumorigenic M2 phenotype to an anti-tumorigenic M1 phenotype. A similar phenotypic switch was seen with pharmacologic antagonism of prostaglandin EP_4_ receptors. Macrophages/dendritic cells, a major component of stroma, normally exhibit a tumor-promoting M2 phenotype in CRC [33], and higher macrophage density in the tumor is associated with poor prognosis [34, 35]. Myeloid cell COX-2-derived PGE2 is essential for promoting the M2/Th2 phenotype seen in infiltrating cells in tumors [36], and COX-2 inhibition has been reported to polarize macrophages/dendritic cell to an anti-tumorigenic, pro-inflammatory M1 (“classically activated”) phenotype [14, 37]. Therefore, myeloid cell PGE_2_-EP_4_ signaling plays an important role in polarization and maintenance of a pro-tumorigenic M2 phenotype for macrophages/dendritic cells.

How does the myeloid EP_4_ receptor induce and maintain macrophages/dendritic cells as a pro-tumorigenic M2 phenotype? In a colon cancer animal model, overexpression of decoy receptor 3 led to enhanced tumor growth in association with an increase in M2 phenotypic macrophages/dendritic cells, which was abolished by antagonism of arginase 1, an inducer and marker of M2 macrophages/dendritic cells [38]. In addition, c-MYC is a key player in macrophage/dendritic cell M2 polarization as well as maintenance of pro-tumorigenic factors such as expression of VEGF, TGF-β, the hypoxia inducible factor 1 α-subunit (HIF-1α), and matrix metalloproteinase 9 (MMP9) [39]. Both adenoma arginase 1 and c-MYC expression levels were markedly reduced in myeloid cell EP_4_^−/−^ *Apc*^Min/+^ mice. Therefore, activation of myeloid cell EP_4_ receptor may induce and maintain macrophages/dendritic cells in an M2 phenotype, at least in part, through induction of arginase 1 and c-Myc.

What are the implications for the present study? Two recent reports showed that regular use of aspirin, which non-selectively inhibits cyclooxygenase activity, not only reduces the incidence of CRC, but also provides a beneficial outcome after diagnosis. Regular aspirin use after the diagnosis of CRC was associated with lower risk of colorectal cancer-specific and overall mortality. However, this beneficial effect was only evident among individuals with tumors that overexpressed COX-2 or among patients with an activating PI3K mutation [40, 41]. Our current study suggests that antagonism of EP_4_ receptor may have a similar beneficial effect due to its ability to inhibit the PI3K signaling pathway.

In summary, this study indicating that inhibition of prostaglandin EP4 receptors effectively inhibits tumorigenesis in *Apc*^Min/+^ mice may represent a novel approach for colorectal cancer chemoprevention/adjunct therapy because of the following advantages: (i) inhibition of EP4 receptors is not expected to incur the cardiovascular events posed by the selective COX-2 inhibitors that suppress COX-2-derived prostacyclin production in vascular endothelial cells; (ii) gastric bleeding and ulcers are the major adverse effects of chronic use of NASIDs due to inhibition of COX-1-derived PGE2 production. The PGE2-mediated gastric cytoprotection is primarily through activation of the EP_1_ receptors [42]. Therefore, selective inhibition of EP_4_ receptors may avoid the potential gastric adverse effects due to COX-1 inhibition incurred by NSAIDs; (iii) inhibition of EP_4_ receptors leads to suppression of the PI3K/AKT/mTOR pathway and subsequent tumor cell proliferation; and (iv) inhibition of EP4 receptors leads to polarization of macrophages/dendritic cells from a tumorigenic M2 phenotype to an anti-tumorigenic M1 phenotype.

## MATERIALS AND METHODS

### Apc^*Min/+*^ mouse model

All animal experiments were performed in accordance with the guidelines and with the approval of the Institutional Animal Care and Use Committee of Vanderbilt University. The germ-line mutations in the adenomatous polyposis coli (*APC*) gene lead to familial adenomatous polyposis, and inactivation of APC is also found in most sporadic colorectal cancers [5]. *Apc*^Min/+^ mice have an autosomal dominant heterozygous nonsense mutation of the mouse *Apc* gene, homologous to human germ-line and somatic *APC* mutations. *Apc*^Min/+^ mice develop adenomas to a grossly detectable size within a few months. Male *Apc*^Min/+^ mice (stock number 002020) were purchased from Jackson Laboratory (Bar Harbor, MA). EP4^flox/flox^ mice were generated in Dr. Breyer's laboratory [43]. CD11b-Cre mice with transgene integration in the Y-chromosome were generated in Dr. Vacher's laboratory [21]. All mouse strains were on the C57BL/6 background.

*Apc*^Min/+^ mice and CD11b-Cre mice were crossed with EP_4_^flox/flox^ mice to generate *Apc*^Min/+^; EP_4_^flox/flox^ mice and CD11b-Cre; EP_4_^flox/flox^ mice, which were intercrossed again to generate EP_4_^flox/flox^; *Apc*^Min/+^ (WT) mice and CD11b-Cre; EP_4_^flox/flox^ (myeloid cell EP_4_^−/−^) *Apc*^Min/+^ mice. Of note, genotypes were re-confirmed after sacrifice at 20 weeks of age. Under anesthesia with Nembutal (60 mg/kg, i.p.), the entire intestine was dissected, flushed thoroughly with ice-cold PBS (pH 7.4), and then filled with fixative [44]. The intestine was transferred to 70% ethanol for 24 h, opened longitudinally, and examined using a dissecting microscope to count polyps in a blinded fashion. The tumor diameter was measured with a digital caliper. After tumors were counted, intestinal tissues were processed for paraffin embedding [45].

A subgroup of myeloid cell EP_4_^flox/flox^; *Apc*^Min/+^ mice at 16 weeks of age was treated with either vehicle (saline) or the selective prostaglandin EP_4_ receptor antagonist, L-161, 982, at a dose of 10 mg/kg/day via daily intraperitoneal injection [46, 47]. One week later, the animals were sacrificed and adenomas were harvested for immunostaining and snap frozen in liquid N_2_ for biochemical analysis.

Genotyping. DNA was isolated from tail snips. Cre and Cox-2 allele genotypes were determined as previously described [48]. A PCR-based protocol from the Jackson Laboratory was adapted to genotype the *Apc* locus. PCR reactions for *Apc* wild type or *Min* alleles were carried out separately with appropriate positive, negative and no template controls. All PCR reactions were carried out using an MJ Research thermal cycler.

### Immunofluorescence/immunohistochemistry staining and quantitative image analysis

Immunostaining was carried out as in previous reports [49]. For both immunofluorescent and immunohistochemical staining of all phosphorylated proteins, antigen retrieval was achieved by boiling in citric acid buffer (100 mM, pH 6.0) for 3 × 5 min. For F4/80 immunofluorescent staining, antigen retrieval was achieved by incubating in trypsin solution for 15 min (T-7186, Sigma). For immunofluorescent staining, deparaffinized sections were blocked with different blocking solutions according to the target of interest for 1 h and then incubated with primary antibodies overnight at 4oC, after washing with PBS, the section was processed as described in Supplemental Table 1. VECTASHIELD mounting medium with DAPI was used for nuclear staining (H-1200, Vector Laboratories). Sections were viewed and imaged with a Nikon TE300 fluorescence microscope and spot-cam digital camera (Diagnostic Instruments). On the basis of the distinctive density and color of immunostaining in video images, the number, size, and position of stained area were quantified by using the BIOQUANT true-color windows system (R & M Biometrics, Nashville, TN), as previously described [45]. Four representative fields from each animal were quantified at x160 magnification, and their average was used as data from one animal sample.

### Immunoblotting

Small intestinal adenomas were homogenized with buffer containing 10 mM Tris·HCl (pH 7.4), 50 mM NaCl, 2 mM EGTA, 2 mM EDTA, 0.5% Nonidet P-40, 0.1% SDS, 100 μM Na3VO4, 100 mM NaF, 0.5% sodium deoxycholate, 10 mM sodium pyrophosphate, 1 mM PMSF, 10 μg/ml aprotinin, and 10 _g/ml leupeptin. The homogenate was centrifuged at 15,000 *g* for 20 min at 4°C. An aliquot of supernatant was taken for protein measurement with a BCA protein assay kit (ThermoScientific, Rockford, IL). Immunoblotting was described in a recent report [12].

### *In situ* hybridization

*In situ* hybridization was performed with digoxigenin-labeled nucleic acid probes as described previously with some modifications [50]. Briefly, the mouse COX-2 gene antisense probe was labeled with DIG RNA labeling kit (Roche Applied Science, Mannheim, Germany) and the sense probe was synthesized at the same time as a control. Mouse kidneys were fixed in 4% paraformaldehyde, then processed to 10 μm paraffin sections. Paraffin slides were fixed in 4% paraformaldehyde for 10 min, deparaffinized and deproteinized with protease K for 15 min. Slides were then fixed again with 4% paraformaldehyde for 5 min. After washing with PBS, slides were acetylated for 10 min, and permeabilized with 1% Triton X-100 for 20 min. After washing with PBS, pre-hybridization was carried out at 55°C for 2 h. Subsequently, slides were incubated in hybridization buffer with probes at 55°C overnight and then washed with 0.2X SSC for 2 h, Tris-saline buffer for 5 min, followed by blocking with 10% heat inactivated sheep serum for 2 h. The probe-target complex was detected immunologically by a digoxigenin antibody conjugated to alkaline phospatase acting on nitro blue tetrazolium chloride/5-bromo-4-chloro-3-indolyl phosphate (NBT/BCIP, Roche Applied Science, Mannheim, Germany) according to the manufacturer's protocol.

### Cell culture

Murine macrophage RAW264.7 cells were grown in DMEM supplemented with 4,500 mg/L glucose, 2 mM L-glutamine, 10% fetal bovine serum, 100 U/ml penicillin, and 100 μg/ml streptomycin in 5% CO_2_ and 95% air at 37°C. The cells were starved for 16 hours in medium containing 0.5% fetal bovine serum. Vehicle (DMSO) or EP4 receptor antagonist, L-161,982 (20 μM dissolved in DMSO) was added 30 min before 10 μM PGE_2_ was added for additional 3 hours. The cells were harvested for qPCR measurements.

### RNA isolation and quantitative real time PCR

Total RNA was isolated from isolated myeloid cells and tumors from *Apc^Min/+^* mice using Trizol reagents (Invitrogen) according to the manufacturer's instructions. Quantitative PCR was performed using the iCycler iQ Real Time PCR detection System (Bio-Rad, Richmond, CA). The following primers were used: prostaglandin EP_4_ receptor (Mm00436053), arginase 1 (Mm00475991), iNOS (Mm00440502), caspase 3 (Mm01195084) and GAPDH (Mm99999915).

### Isolation of intestinal monocytes/macrophages/dendritic cells

CD11b-expressing cells in intestine single cell suspensions were enriched using mouse CD11b Microbeads and MACS columns (Milteni Biotec Auburn, CA) following the manufacturer's protocol.

### Antibodies

The primary antibodies that were used for immunohistochemistry and immunoblotting included rabbit anti-mouse COX-2 from Cayman Chemicals, cyclin D1 and c-Myc from Santa Cruz Biotechnology; rat anti-mouse F4/80 (marker of macrophages/dendritic cells) from AbD Serotec; rabbit anti-p-ERK, p-p70 S6K (Thr389), p-PI3K p85 (Tyr458), p-PDK1 (Ser241), p-AKT (Thr308), p-mTOR (Ser2448), p-raptor (Ser792), p-elF-4B(Ser422), p-S6 ribosomal protein (p-rpS6, Ser240/244), and rabbit anti-cleaved caspase-3 (9661) from Cell Signaling Technology; rabbit anti-iNOS, rabbit-anti-Ki67 (ab15580), and goat anti-arginase 1 from Abcam, mouse anti-interleukin 4 receptor α (IL-4Rα) and mannose receptor (MR, CD206) from R&D.

### Statistics

All values are presented as means, with error bars representing ± s.e. Fisher exact test, analysis of variance (ANOVA) and Bonferroni *t* tests were used for statistical analysis.

## SUPPLEMENTARY MATERIAL FIGURES AND TABLE


