# Supplementary material for: Prostaglandin E receptor 4 (EP4) promotes colonic tumorigenesis
Source: Oncotarget. 2015 Sep 10;6(32):33500–11. doi: 10.18632/oncotarget.5589 (PMC4741781; doi:10.18632/oncotarget.5589)
Supplement: Supplementary file 1 [file oncotarget-06-33500-s001.pdf]

Prostaglandin E receptor 4 (EP4) promotes colonic tumorigenesis

Supplementary Material

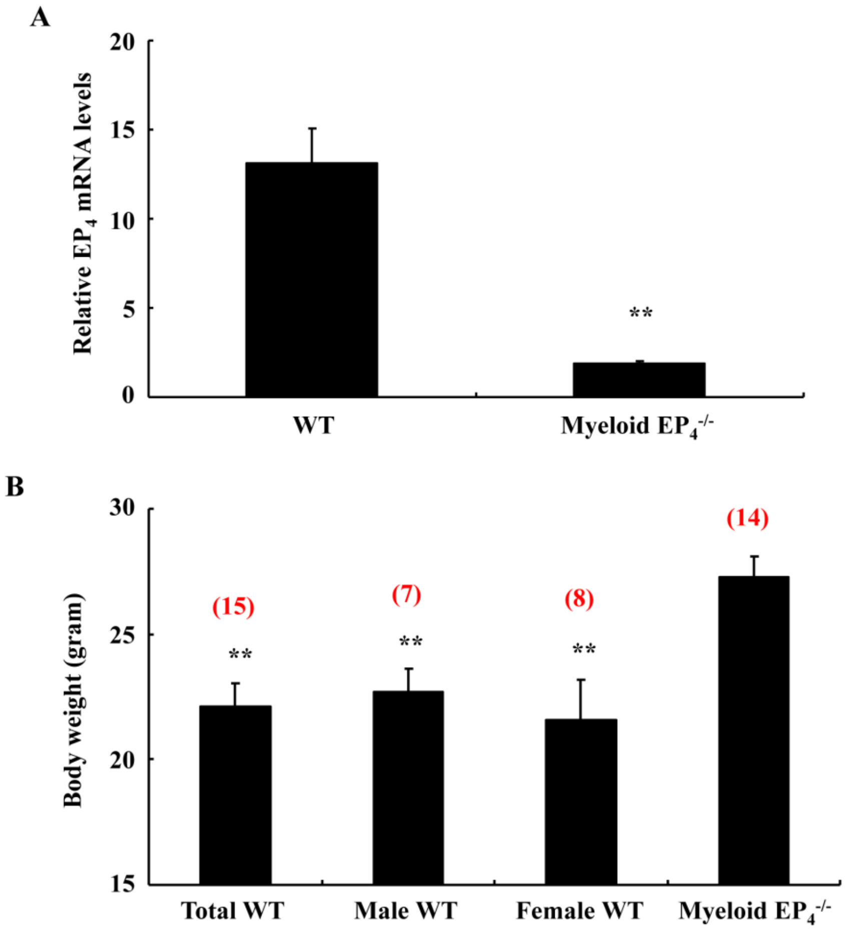

Figure S1. Deletion of myeloid cell EP_4 receptors was associated with higher body weight in *Apc*$^{Min/+}$ mice. **A**: EP_4 mRNA levels were markedly reduced in isolated intestinal myeloid cells in myeloid cell $EP_4^{-/-}$ *Apc*$^{Min/+}$ mice compared with wild type *Apc*$^{Min/+}$ mice (**P < 0.01, n = 4). **B**: Body weight was significantly higher in myeloid cell $EP_4^{-/-}$ *Apc*$^{Min/+}$ mice than in male or female or total wild type *Apc*$^{Min/+}$ mice (**P < 0.01). Animal number is indicated in the brackets.

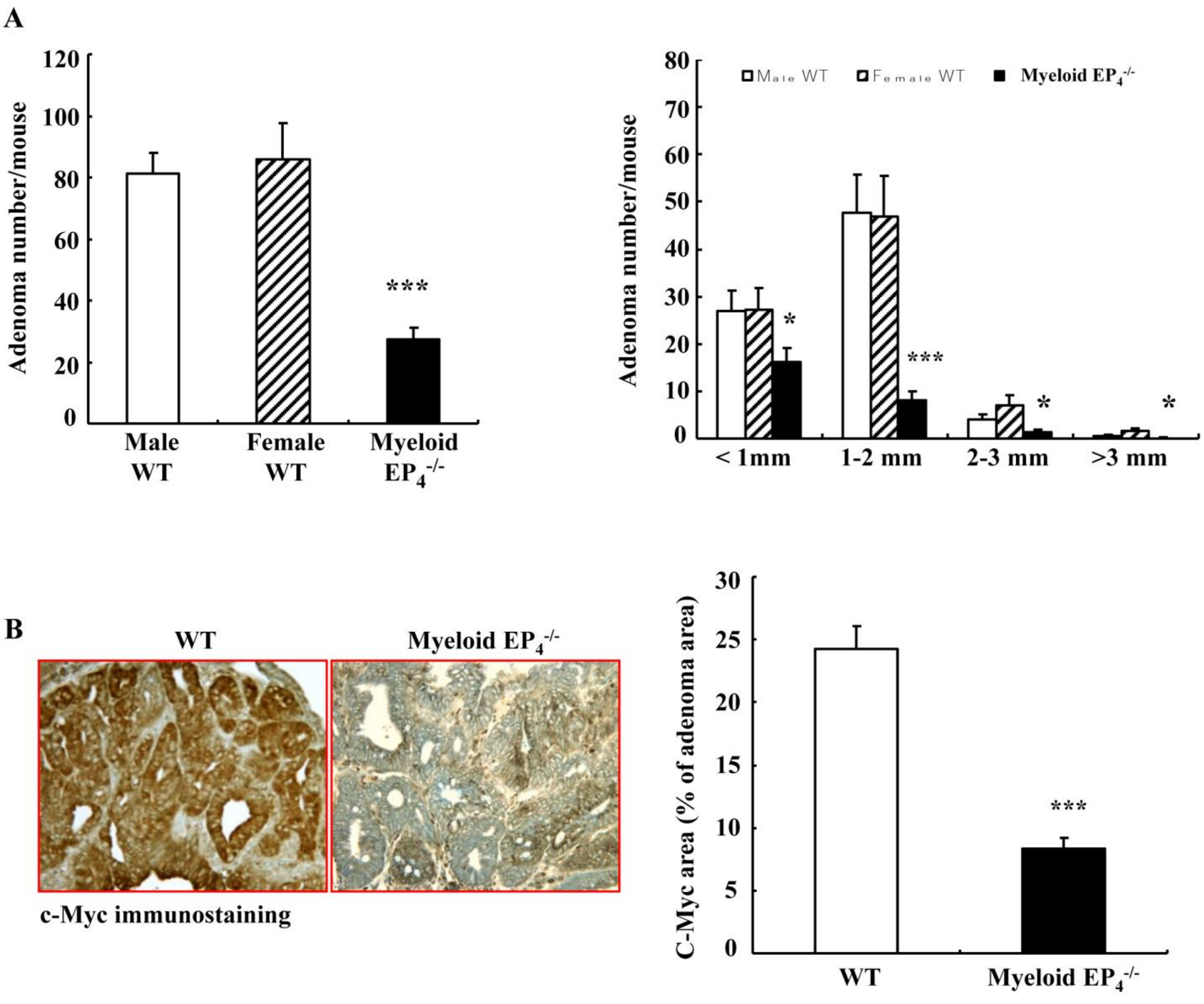

Figure S2. No gender difference was found in tumorigenesis in $EP_4^{flox/flox}$ (wild type) *Apc*$^{Min/+}$ mice. **A**: Both intestinal adenoma multiplicity and sizes were similar between male and female wild type *Apc*$^{Min/+}$ mice, but decreased in myeloid cell $EP_4^{-/-}$ *Apc*$^{Min/+}$ mice (**P* < 0.05, ***P* < 0.01, ****P* < 0.001 vs. male or female WT, n = 7 in male WT group and n= 8 in female WT group). **B:** Immunostaining indicated that c-Myc was primarily expressed in nuclei and cytosol in adenoma epithelial cells and its expression decreased in myeloid cell $EP_4^{-/-}$ *Apc*$^{Min/+}$ mice (****P* < 0.001, n = 4 in each group). Original magnification: x160.

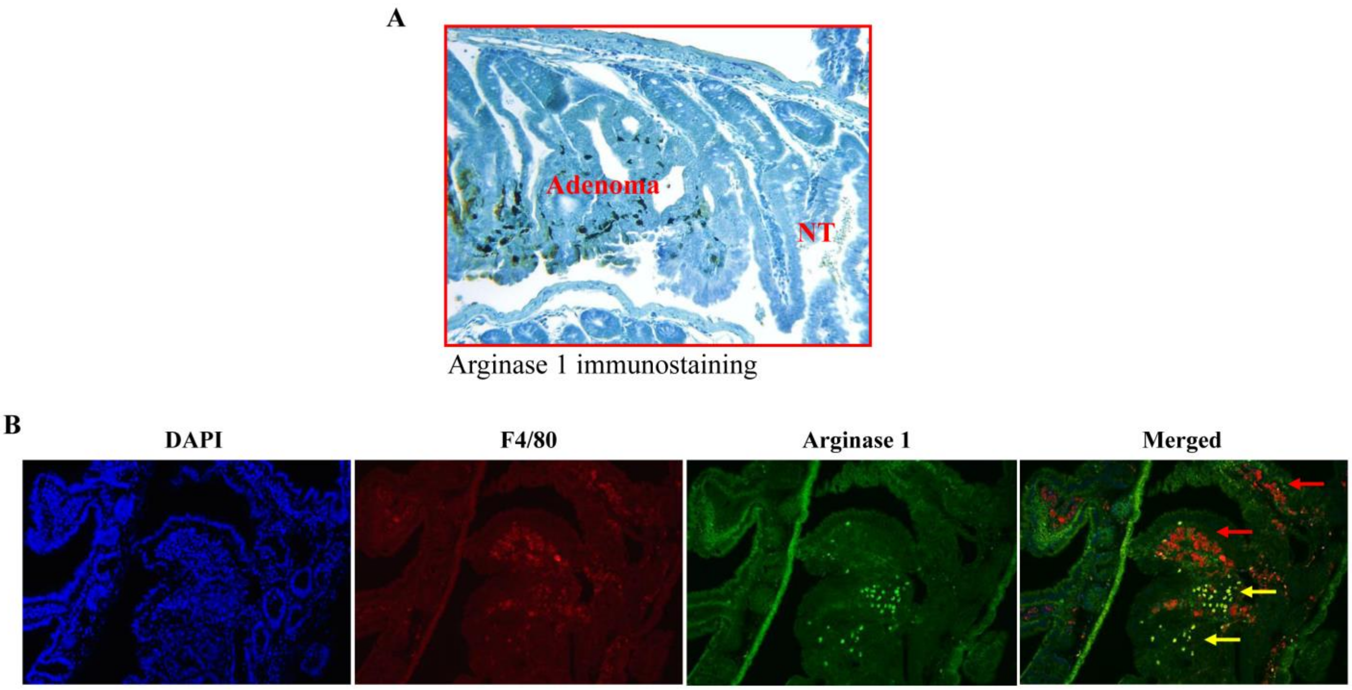

Figure S3. Macrophages/dendritic cells exhibited differential phenotypes in normal intestine and in adenoma in wild type *Apc*$^{Min/+}$ mice. **A**: Arginase 1, a commonly used marker of M2 macrophages/dendritic cells, was expressed in many tumor stromal cells but was not detected in normal intestine tissue (NT). **B**: Double fluorescent staining indicated that arginase 1 was expressed in most macrophages/dendritic cells (yellow arrows), but rarely expressed in macrophages/dendritic cells (red arrows) in adjacent normal intestinal tissue in wild type *Apc*$^{Min/+}$ mice. Original magnification: x 100 in all.

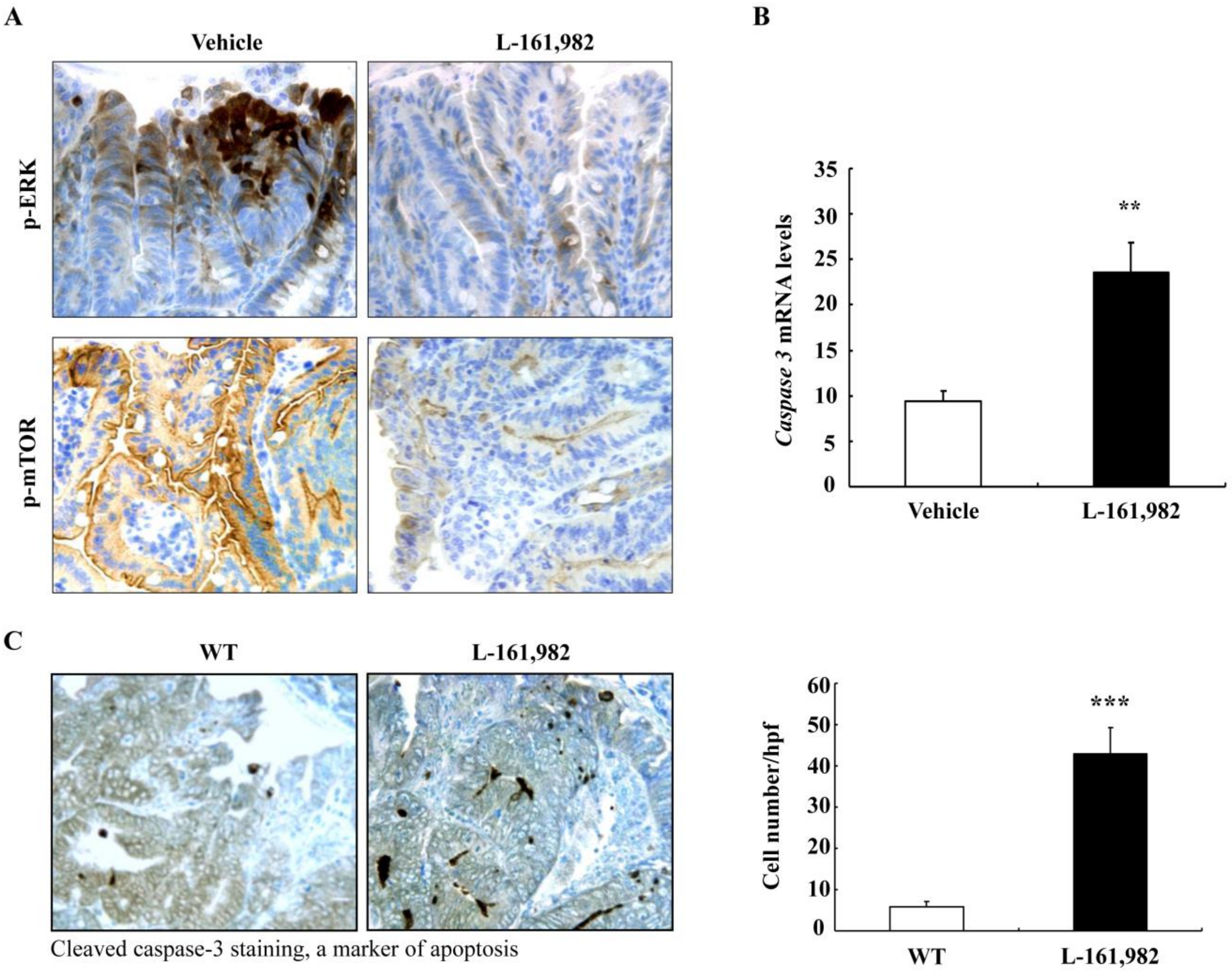

Figure S4. Pharmacologic inhibition of EP_4 receptors suppressed adenoma activities of the ERK and mTOR pathways and induced apoptosis. **A**: Treatment with L-161,982, a selective EP_4 receptor antagonist for a week led to decreased phosphorylation of ERK and mTOR. Original magnification: x160. **B**: L-161,982 treatment led to increased mRNA levels of caspase 3, indicating induction of apoptosis (*** *P* <0.01 vs. vehicle, n = 5). C. L-Treatment with 161,982 led to increases in adenoma apoptotic cells as indicated by cleaved caspase-1 staining (*** *P* <0.01 vs. vehicle, n = 4). Original magnification: x250.

Supplemental Table 1. Procedure for double fluorescent staining

Antibody	Blocking	1st Ab Host	Detection
COX-2	10% donkey serum	rabbit	Donkey anti-rabbit IgG-FITC
Arginase 1	10% donkey serum	goat	Donkey anti-goat IgG-FITC
IL-4Rα	M.O.M kit	mAb	Goat anti-mouse IgG-FITC
F4/80	10% goat serum	rat	Biotin-goat anti-rat IgG plus Texas red streptavidin

Note: M.O.M kit: for detecting mouse primary antibodies on mouse tissue peroxidase.
